# Analysis and prediction of the temporal and spatial evolution of carbon emissions in China’s eight economic regions

**DOI:** 10.1371/journal.pone.0277906

**Published:** 2022-12-01

**Authors:** Zhen Yu, Yuan Zhang, Juan Zhang, Wenjie Zhang

**Affiliations:** 1 Institute of Oceanographic Instrumentation, Qilu University of Technology (Shandong Academy of Sciences), Qingdao, China; 2 School of Management, China University of Mining & Technology (Beijing), Beijing, China; 3 Airport College, Binzhou University, Binzhou, China; 4 School of Economics and Management, Southeast University, Nanjing, China; Instituto Tecnologico Autonomo de Mexico, MEXICO

## Abstract

Facing increasingly severe environmental problems, as the largest developing country, achieving regional carbon emission reduction is the performance of China’s fulfillment of the responsibility of a big government and the key to the smooth realization of the global carbon emission reduction goal. Since China’s carbon emission data is updated slowly, in order to better formulate the corresponding emission reduction strategy, it is necessary to propose a more accurate carbon emission prediction model on the basis of fully analyzing the characteristics of carbon emissions at the provincial and regional levels. Given this, this paper first calculated the carbon emissions of eight economic regions in China from 2005 to 2019 according to relevant statistical data. Secondly, with the help of kernel density function, Theil index and decoupling index, the dynamic evolution characteristics of regional carbon emissions are discussed. Finally, an improved particle swarm optimization radial basis function (IPSO-RBF) neural network model is established to compare the simulation and prediction models of China’s carbon emissions. The results show significant differences in carbon emissions in different regions, and the differences between high-value and low-value areas show an apparent expansion trend. The inter-regional carbon emission difference is the main factor in the overall carbon emission difference. The economic region in the middle Yellow River (ERMRYR) has the most considerable contribution to the national carbon emission difference, and the main contributors affecting the overall carbon emission difference in different regions are different. The number of regions with strong decoupling between carbon emissions and economic development is increasing in time series. The results of the carbon emission prediction model can be seen that IPSO-RBF neural network model optimizes the radial basis function (RBF) neural network, making the prediction result in a minor error and higher accuracy. Therefore, when exploring the path of carbon emission reduction in different regions in the future, the IPSO-RBF neural network model is more suitable for predicting carbon emissions and other relevant indicators, laying a foundation for putting forward more scientific and practical emission reduction plans.

## 1. Introduction

The impact of global warming is omnidirectional, multi-scale, and multi-level. Its impact scope can be roughly divided into the natural ecological environment and human social development. Global climate change is closely related to the sustainable development of all countries globally. Facing the global warming trend, all countries worldwide are actively exploring ways to curb the further deterioration of climate [1]. As the most critical manufactured greenhouse gas, the sharp increase in carbon dioxide concentration is the main factor leading to global warming. Therefore, the research on the emission reduction of greenhouse gases such as carbon dioxide has become the main content of countries worldwide to explore and curb the trend of global warming [2]. On September 22, 2020, Chinese President Xi Jinping delivered an important speech at the General Debate of the seventy-fifth Session of the United Nations General Assembly, announcing that China would adopt more effective policies and measures to make China’s CO_2_ emissions reach the peak before 2030 and achieve carbon neutrality before 2060 (referred to as "double carbon goals") [3]. Therefore, energy conservation and emission reduction are essential strategic issues that China and the world will pay attention to in the future.

At present, domestic research on carbon emissions mainly focuses on the following aspects: (1) Accounting and prediction of total carbon emissions. (2) Analysis of driving factors of carbon emissions. (3) Regional energy carbon emission pattern and prediction [4, 5]. (4) Carbon emission reduction technology evaluation and policy analysis. Internationally, the research focuses on calculating and predicting greenhouse gas emissions and regional energy conservation and emission reduction [6, 7]. Although domestic and foreign academia has conducted relevant research on carbon emission prediction, with the determination of China’s dual carbon goals, the research on more accurate carbon emission prediction model becomes more important. Wu and Liu et al. predicted the carbon dioxide emissions of BRICS (Brazil, Russia, India, China, and South Africa) countries in 2015 and 2020 by considering the rolling multivariable grey prediction model of energy consumption and economic growth [8]. Ding and Dang et al. improved the traditional grey multivariable model. The results show that the prediction results obtained by the new model are more accurate than the four benchmark models and predicted the carbon dioxide emissions from fuel combustion in China from 2014 to 2020 [9]. Xu and Ding developed the adaptive grey model and combined it with the buffer rolling method to improve accuracy. Compared with the traditional model, they improved the data characteristics’ adaptability and predicted China’s energy greenhouse gas emissions from 2017 to 2025 [10]. Gao and Yang et al. used a novel fractional grey Riccati model (FGRM (1,1)) model to estimate and predict the carbon dioxide emissions of the United States, China, and Japan. They found the best estimation effect [11]. Ren and Long constructed a Fast Learning Network (FLN) forecasting algorithm improved by Chicken Swarm Optimization (CSO) to predict carbon emissions in 2020–2060 Guangdong [12]. Phdungsilp analyzed the historical trend of energy demand and energy-related carbon dioxide emissions in Bangkok, Thailand, and predicted the carbon emissions of four departments in Bangkok by using a Long-range Energy Alternative Planning (LEAP) system [13]. Gao and Yang estimated and predicted the carbon emission of the American industrial sector based on Gompertz law and the fractional grey model. Their results show that the policy change of the United States has an apparent promoting effect on American industrial carbon emission [14]. Sun and Ren first applied ensemble empirical mode decomposition (EEMD) to the field of carbon emission prediction based on particle swarm optimization back propagation neural network (PSOBP), which improved the accuracy of prognosis [15]. Yan and Liu et al. studied the prediction of land carbon emissions based on the principal component analysis back propagation (PCA-BP) neural network, which provided a quantitative basis for urban low-carbon planning and carbon emission control [16]. Taking Xi’an as an example, Hu and Gong predicted the carbon emission of urban household consumption in Western China based on the back propagation (BP) neural network model, which provided new ideas and guidance for accurately identifying the carbon emission prediction of urban household consumption [17]. Fan and Zhang predicted the total amount and intensity of carbon emissions from 30 provinces and cities in China from 2020 to 2030 by establishing a PSO-BP neural network model [18]. Qiu and Cai combined with BP neural network model to predict the carbon emission value of Shaanxi and reasonably analyze the expected value and the changing trend of carbon emission. They put forward the solutions to the related problems of carbon emission reduction [19]. Yang and Liang et al. used the optimized Radial Basis Function (RBF) model to predict the carbon price in the EU carbon emission trading market, making the model’s prediction accuracy more accurate [20]. LV and Hu used a grey quantum particle swarm optimization available vector machine to predict and study the carbon emission transfer of 28 industries in China. They constructed an inter-industry carbon emission transfer network [21].

In terms of existing research methods, RBF neural network has apparent advantages over BP neural network in terms of generalization ability, approximation accuracy, learning rate, etc. Although RBF neural network has the advantage of approximating any functions with arbitrary accuracy, its structure is complex, its convergence speed is slow, the amount of computation is large, and it is easy to fall into the local optimum, and it even may not converge. Therefore, in order to avoid the above problems, this paper adopts IPSO-RBF neural network model, which first optimizes the weights and learning factors in the PSO algorithm, and then applies the improved new particle swarm optimization algorithm to the determination of RBF neural network parameters. It realizes the optimization of the center value and width vector of the hidden layer Gaussian function of RBF neural network and the weights between the hidden layer and the output layer, and improves the prediction accuracy of RBF neural network.

The marginal contribution of this paper is to find out the change trend of carbon emissions in different regions through detailed analysis of the space-time evolution characteristics and regional differences of China’s carbon emissions from the provincial and regional levels, so as to formulate targeted emission reduction measures for carbon emissions in different regions. In order to ensure the realization of China’s dual carbon goals, this paper forecasts the carbon emission data from 2016 to 2019 based on the relevant data of carbon emissions from 2005 to 2015 and in combination with different carbon emission prediction models. By comparing with the actual carbon emission data, the carbon emission prediction model with the highest accuracy is found, which lays a model and data foundation for the prediction of carbon emissions of different provinces and regions in 2030 and 2060. The innovation of this paper is that it not only makes a detailed analysis of the spatio-temporal evolution characteristics and regional differences of regional carbon emissions from the provincial and regional levels, but also introduces a more accurate carbon emissions prediction model based on the full analysis of the change trend of carbon emissions in different regions, laying a good foundation for the prediction of future carbon emissions in different provinces and regions under the dual carbon goals.

## 2. Materials and methods

### 2.1 Regional division standard

Due to China’s vast territory, different provinces differ significantly in terms of development level, environmental conditions, energy mix and industrial structure. These significant differences also lead to substantial differences in the current situation of carbon emissions in various regions. Therefore, whether the spatial and temporal evolution characteristics of carbon emissions in different provinces can be accurately identified is the key to achieving China’s carbon peak by 2030 and carbon neutrality by 2060. However, suppose carbon emission reduction measures are formulated only according to the carbon emissions of different provinces. In that case, it will increase the decision-making cost and be detrimental to realizing the overall national carbon emission reduction target. Therefore, some scholars divided China into three regions: eastern, central and western regions to analyze regional differences in carbon emissions [22]. However, this method of regional division is relatively rough. Therefore, according to the joint regional development strategy and policy report of the Development Research Center of the State Council, this paper takes 30 provinces in China as the research object, and divides them into eight comprehensive economic regions with similar economic development levels. The division basis mainly includes the following points: keep the spatial distance close, the regional economic development level is relatively close, the social structure is relatively similar, the regional scale included in the region is similar, there are reasonably similar development problems, and try to maintain the integrity of the administrative area.

**Fig 1:**
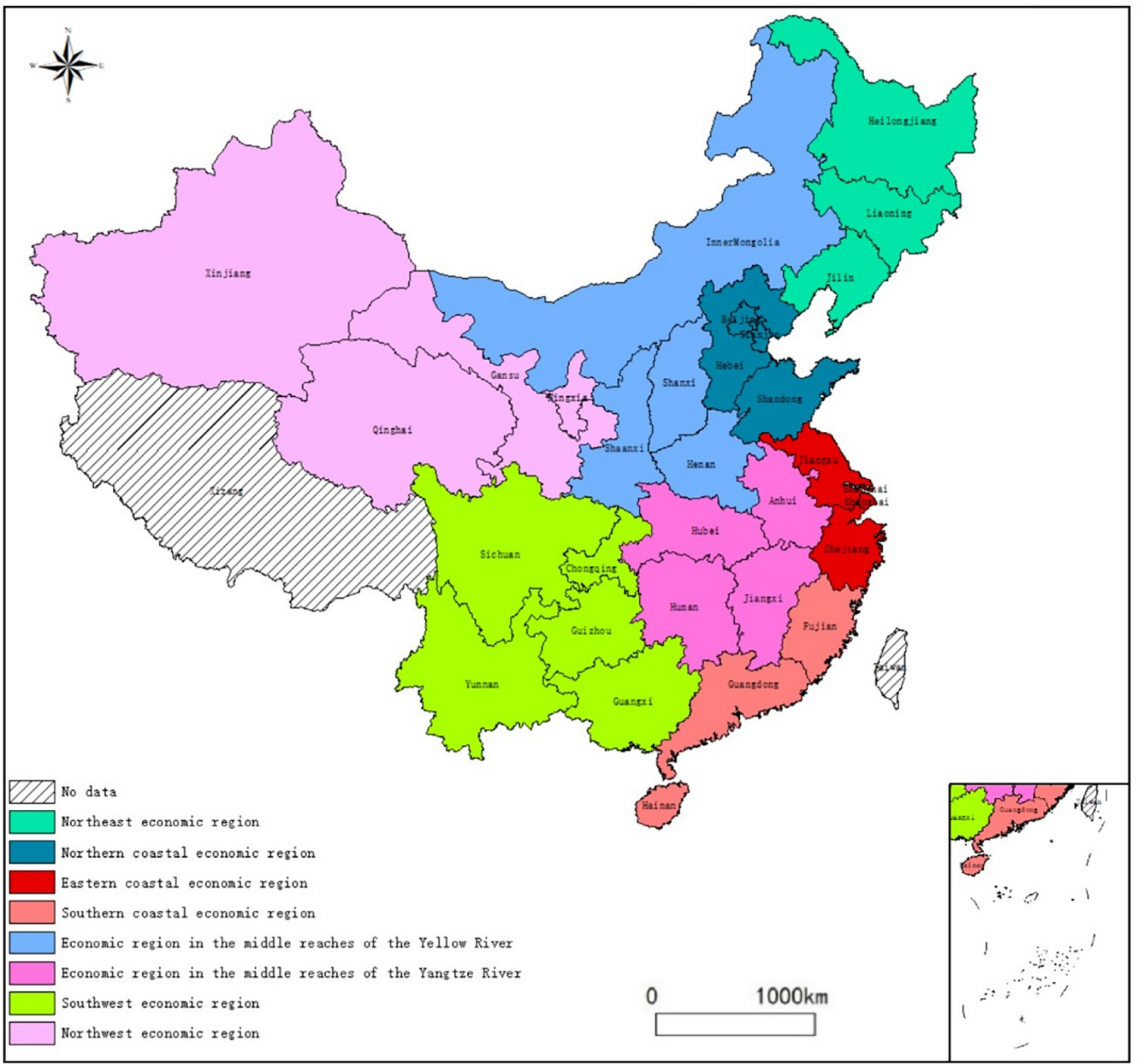
Detailed information on China’s eight economic regions. (Note: The original picture is from Natural Earth, http://www.naturalearthdata.com).

In this paper, in addition to Hong Kong, Macao, Taiwan, and Tibet, China’s 30 provinces are divided into eight economic regions: the Northeast economic region (NEER), the Northern coastal economic region (NCER), the Eastern coastal economic region (ECER), the Southern coastal economic region (SCER), the Economic region in the middle Yellow River (ERMRYR), the Economic region in the middle Yangtze River (ERMRYTR), the Southwest economic region (SWER) and the Northwest economic region (NWER). The specific division details are shown in Fig 1.

Given the slow update of official carbon emission data and the difficulty of traditional prediction models to meet the actual needs, this paper uses numerical simulation statistical data to calculate China’s carbon emissions from 2005 to 2019 through the method recommended by IPCC [23]. Referring to the IPCC guidelines for national greenhouse gas emission inventories 2006, this paper uses the "bottom-up" carbon emission coefficient method to calculate the carbon emissions of different regions, and the formula is ${CE}_{ij}=\sum_{i=1}^{19}A_{ij}\times {NCV}_{j}\times {EF}_{j}\times O_{j}(1)$, where i represents the ith region, j means the jth fuel, where j = 1, 2, 3… 19, describes 19 types of energy such as coal, oil, natural gas, heat, electricity and so on. CE_ij_ refers to the carbon emission of the j fuel in region i. A_ij_ is the final consumption of the jth fuel in the ith region. EF_j_ is the CO_2_ emission coefficient of fuel j. NCV_j_ and O_j_ represents the calorific value and oxidation rate of the jth fuel, respectively.

### 2.2 Kernel density function

According to a specific sample set, parametric and nonparametric estimations are used to obtain its distribution function. The former needs to add subjective factors in advance, so the fitting effect is relatively poor. The latter only needs to fit its distribution density function according to the characteristics of the data itself, and the result is better. Therefore, the nonparametric estimation method with better fitting effect is selected in this paper. The standard nonparametric estimation method is kernel density function, and it has been widely used in various fields [24–27]. The kernel density function describes the distribution state of regional carbon emissions with a continuous density curve. This paper analyzes carbon emission bias distribution characteristics in China’s eight economic regions and the temporal and spatial evolution trend with time and space through the distribution position, shape, and flexibility of carbon emission bias in the nuclear density estimation results. Among them, the position of the Kernel density curve reflects the degree of carbon emission deviation. The width and height of wave crest reflect the degree of dispersion and aggregation of carbon emission deviation between regions. And the number of wave crests reflects the degree of polarization [28]. Distribution ductility indicates the regional difference between the region with the highest carbon emissions and other regions. The longer the tail, the more significant the regional carbon emission difference. Based on previous studies, this paper uses the kernel density function to fit the carbon emissions of the eight economic regions, obtains its probability distribution curve, and then analyzes the evolution track of carbon emissions in this region. The specific calculation method is as follows:

$$f(p)=\frac{1}{\mathrm{nh}}\sum_{i=1}^{n}g(\frac{p-p_{i}}{h})$$ (1)

Where f(p) represents the kernel density estimator, n represents the number of observations in the observation area, p represents the independently distributed observations, p_i_ is the average observed value, g is the kernel function, and h is the bandwidth. The smaller the bandwidth, the more accurate the kernel density estimation is. g(X) represents kernel density function, which is essentially a weight function. The most commonly used kernel function is the quadratic kernel Gaussian kernel function [29]. In this paper, the Gaussian kernel function is adopted, and the expression is:

$$g(X)=\frac{1}{\sqrt{2\pi }}exp(\frac{-X^{2}}{2})$$ (2)

### 2.3 Theil index

The Theil index was first proposed in 1967 and was initially used to calculate the income difference between countries [30]. Later, it was widely used in the income difference of different regions. Its advantage is that it can not only make statistics on the change trend and fluctuation of inter-regional and intra-regional differences, but also reveal its impact on the overall differences. The value range of the Theil index is [0,1]. The closer the value is to 0, the smaller the regional difference. The closer the value is to 1, the more significant the regional difference [31]. Based on previous studies, the Theil index and its structure of regional carbon emissions are decomposed as follows:

$$T=T_{BR}+T_{WR}=\sum_{i=1}^{n}\frac{C_{i}}{C}\ln\left(\frac{C_{i}/C}{G_{i}/G}\right)+\sum_{i=1}^{n}\frac{C_{i}}{C}\left[\sum_{j=1}^{m}\frac{C_{\mathrm{ij}}}{C_{i}}\ln\left(\frac{C_{\mathrm{ij}}/C_{i}}{G_{\mathrm{ij}}/G_{i}}\right)\right]$$ (3)

$$T_{BR}=\sum_{i=1}^{n}\frac{C_{i}}{C}\ln\left(\frac{C_{i}/C}{G_{i}/G}\right)$$ (4)

$$T_{WR}=\sum_{i=1}^{n}\frac{C_{i}}{C}\left[\sum_{j=1}^{m}\frac{C_{\mathrm{ij}}}{C_{i}}\ln\left(\frac{C_{\mathrm{ij}}/C_{i}}{G_{\mathrm{ij}}/G_{i}}\right)\right]$$ (5)

Where T represents the overall Theil index of regional carbon emissions, T_BR_ and T_WR_ represents the inter-regional difference and intra-regional difference of regional carbon emission, respectively, C, C_i_, and C_ij_ refers to the carbon emissions of the whole country, region i, and each province j in region i, G, G_i_, and G_ij_ respectively refers to the GDP of the whole country, region i, and each province j in region i.

### 2.4 Decoupling model between carbon emission and economic growth

Decoupling of carbon emissions refers to the relationship between the change in CO_2_ emissions and economic growth. When economic growth is achieved, the growth rate of CO_2_ emissions is negative or less than the economic growth rate, which can be regarded as decoupling. Its essence is to measure whether economic growth is at the cost of resource consumption and environmental damage. Decoupling carbon emissions is an ideal process. In this process, the relationship between economic growth and greenhouse gas emissions is weakening or even disappearing, and energy consumption is gradually reduced on the basis of economic growth. The elasticity between carbon emissions and economic growth in different regions is an important indicator to measure regional decoupling status, and also a major tool to measure regional low-carbon status.

Since the economic growth of China’s eight major economic regions was positive during the study period, their decoupling status only included four states: strong decoupling (SD), weak decoupling (WD), and expansionary link (EC) and expansionary negative decoupling (END). The specific division details are shown in Table 1, and the calculation formula is as follows:

$$\delta_{ij}=\frac{\Delta {CO}_{2_{ij}}\%}{\Delta {GDP}_{ij}\%}$$ (6)

Where, *δ*_*ij*_ represents the decoupling elasticity value of *i* province in *j* region, $\Delta {CO}_{2_{ij}}\%$ and ΔGDP_ij_% represents the growth of carbon emissions and GDP of each region in the three research periods. The above data of carbon emissions are calculated from Formula 1. The GDP is based on 2005, excluding the influencing factors of price.

**Table 1:** Division basis of decoupling status.

Decoupling status	Abbreviation	ΔGDP (%)	ΔCO2(%)	Value range
Strong decoupling	SD	>0	<0	(−∞, 0)
Weak decoupling	WD	>0	>0	(0,0.8)
Expansionary connection	EC	>0	>0	(0.8,1.2)
Expansionary negative decoupling	END	>0	>0	(1.2, +∞)

### 2.5 IPSO-RBF neural network model

**Fig 2:**
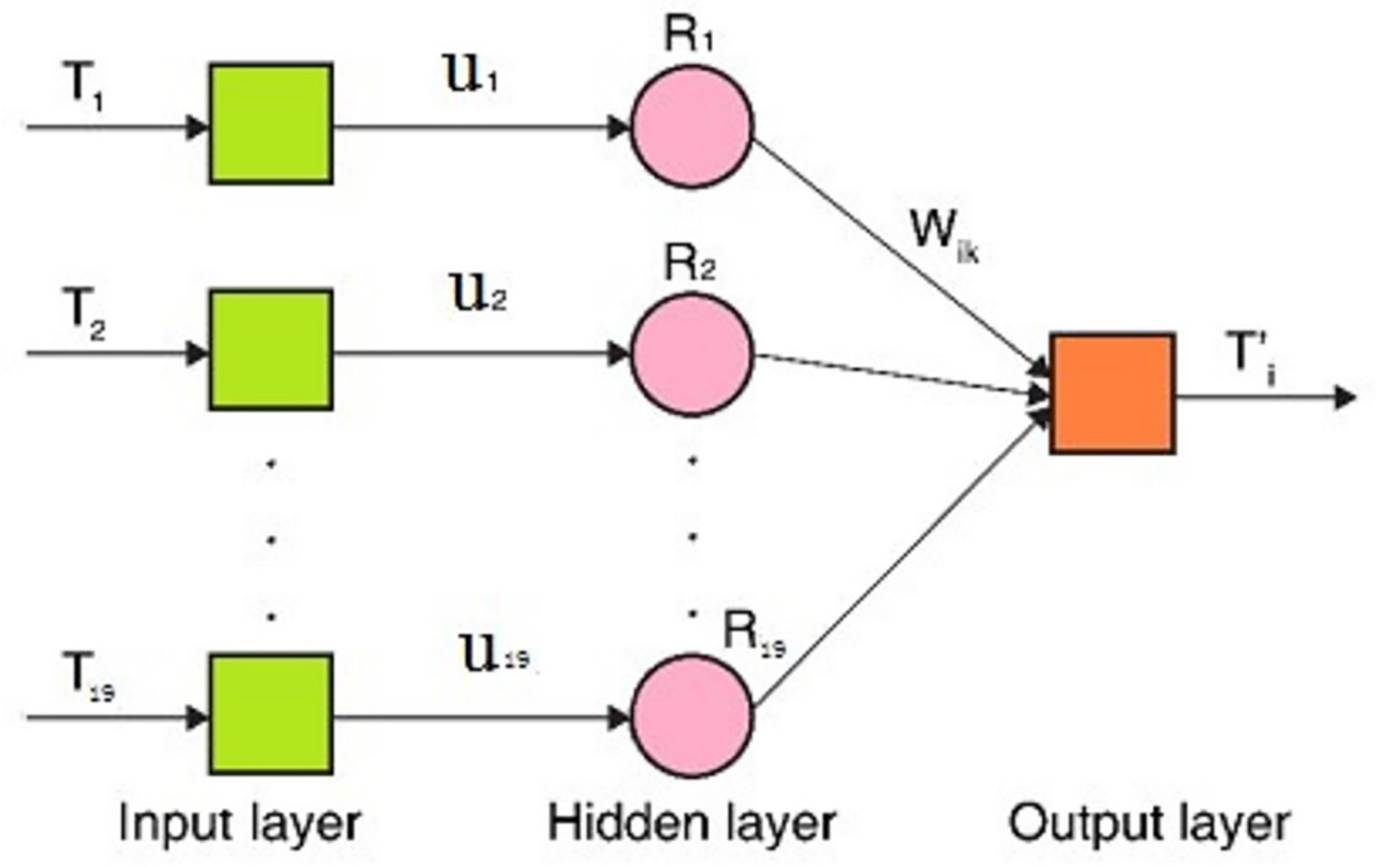
Error compensation model.

Supposing that the relationship between energy consumption and carbon emissions can be formulated according to the 11 groups of scattered points, which are the data from 2005 to 2015, the future carbon emissions can be obtained using the relationship and the energy consumption. Therefore, the RBF neural network is used to realize continuous compensation, which can approximate any function with an arbitrary accuracy [32]. Multitudinous scholars have proved that using an RBF neural network is better than general compensation methods, such as polynomial fitting and the BP neural network [33]. As shown in Fig 2, the RBF neural network for the error compensation is a three-layer unidirectional propagation network composed of input, output, and hidden layers. There are 19 nodes in the network’s input layer, which uses the 19 energy consumption categories, such as coal, oil, natural gas, heat, and electricity, represented by T_i_. There is one node in the output layer, which uses the carbon emissions, described as T’_i_. The number of nodes in the hidden layer is automatically calculated and set by the Newrbe function. Repeated debugging showed that for 19 hidden-layer nodes, the RBF neural network converges the fastest, and the fitting effect is optimal. Therefore, the number of hidden-layer nodes was set as 19, with the i-th node represented by R_i_. The weight from T_i_ to R_i_ is u_ik_ based on the relationship between energy consumption and carbon emissions, as expressed in Eq (7). The hidden layer unit is transformed by the radial basis function and sent to the output layer. The radial basis function is the Gaussian basis function. The weight from R_i_ to T’_i_ is W_ik_, using a linear function as an output layer function.

The energy consumption and the carbon emissions are used as the learning samples for training. The hidden layer output is expressed as follows:

$$R_{i}=e^{-\frac{\left\|u_{ik}T_{i}-c_{i}\right\|}{2\sigma_{i}^{2}}}=e^{-\frac{\left\|\sum_{i=1}^{17}A_{ij}\times {NCV}_{i}\times {EF}_{i}\times O_{i}-c_{i}\right\|}{2\sigma_{i}^{2}}}$$ (7)

In the equations above, c_i_ is the center of the i-th node, σ_i_ is the parameter controlling the receiving domain size, and ‖·‖ is the Euclidean Norm. The hidden layer training calculates the values of the center c_i_ and width σ_i_ calculated in the invisible layer training. The model of the output layer node is expressed as follows:

$${T\prime }_{i}=\sum_{k=1}^{50}W_{ik}R_{i}$$ (8)

In the equations above, *W*_*ik*_ denotes the output weights from R_i_ to T’_i_. The weights are calculated in the training of the last layer. The total error-calculation function is expressed as follows:

$$e=\sum_{i=1}^{n}\sum_{k=1}^{50}{\left({T\prime }_{ik}-t_{ik}\right)}^{2}$$ (9)

Here, t_ik_ denotes the calculation result of the output layer node. The structure and RBF neural network parameters can be acquired by adjusting the weights and thresholds. The trained RBF neural network can be used to estimate future carbon emissions.

The RBF neural network parameters, c_i_, σ_i_, and W_ik_ must be determined through learning and training. The IPSO algorithm is used to optimize the network parameters of the RBF neural network used in this study. The model optimization algorithm described in this paper evolved from the particle swarm optimization (PSO) algorithm [34]. The PSO algorithm simulates bird clusters’ flight and foraging behavior and makes the group achieve its goal through cooperation between birds. In the PSO algorithm, each particle represents a solution of the cubic spline equation. Among all solutions, the best solution (with the smallest residual) is the best position, and each particle will find the best position in this region. In finding the best place, each particle will find the position closest to the best place, called the individual extremum. The best position of all particles in the search process is the global extremum. These particles will constantly adjust their speed and direction to approach the best position through these two positions. The updated formula of particle velocity and function is as follows.


$$v_{ij}^{m+l}=wv_{ij}^{m}+c_{1}r_{1}(p_{ij}^{m}-x_{ij}^{m})+c_{2}r_{2}(g_{j}^{m}-x_{ij}^{m})$$ (10)

$$x_{ij}^{m+l}=x_{ij}^{m}+lv_{ij}^{m+l}$$ (11)

In the equations above, i = 1, 2, …, n, j = 1, 2, …, j, m = 1, 2, …, M. m is the number of iterations, $x_{ij}^{m}$ is the position of particle i in space, $v_{ij}^{m}$ is the velocity of particle i in space, $p_{ij}^{m}$ and $g_{j}^{m}$ are defined as individual extremum and global extremum, respectively. c1 and c2 are acceleration coefficients, usually c_1_ = c_2_. r_1_ and r_2_ are random numbers in the interval [0,1], and w is the weight.


$$w=w_{max}-(w_{max}-w_{min})\times m/m_{max}$$ (12)

In the equation above, m_max_ is the maximum number of iterations, w_max_ and w_min_ represents the maximum and minimum weights, respectively.

The realization steps of the IPSO-RBF model include three steps: neural network construction, training, and prediction. We take the data from 2005 to 2015 as the training sample, the data from 2016 to 2019 as the test simulation sample, and the data from future carbon emissions, such as 2020, 2030, 2060, etc as the prediction target sample. The training is mainly to assign the optimal weight and threshold obtained by the IPSO algorithm to the RBF neural network as the initial weight and point of the network. The training and test samples are substituted into the network for training and testing. If the actual output of 100 test samples is consistent with the expected result, it indicates that the network’s generalization ability is good and the training is completed. Finally, the carbon emissions of each region in different years in the future are predicted. The above three-step process can be realized using the newff function, train function, and sim function provided by the neural network toolbox in MATLAB 2020b.

## 3. Results and discussion

### 3.1 Carbon emission measurement results

Calculate the carbon emissions of different provinces from 2005 to 2019 according to Formula 1. Based on the basic principle of spatial differentiation and combined with the natural discontinuity classification method in ArcGIS 10.2 software, 30 provinces in China are divided into five categories (lowest, lower, medium, higher, and highest emission areas) to analyze the spatial differentiation pattern of regional carbon emission. The relevant results are shown in Table 2.

**Table 2:** Carbon emission estimation results of each province in 2005, 2010, 2015, and 2019. (Unit:10^4^t).

Region	2005		2010		2015		2019		Mean value	
	CE	Partition	CE	Partition	CE	Partition	CE	Partition	CE	Partition
Beijing	7016.37	2	7437.03	2	7015.32	1	6441.17	1	7284.89	2
Tianjin	5444.72	2	8038.52	2	6693.81	1	10322.91	2	8246.51	2
Hebei	32999.81	5	43463.33	5	48076.43	5	49313.42	5	45222.83	5
Shanxi	18267.03	4	23063.72	4	24306.43	4	23761.72	4	23259.71	4
Inner-Mongolia	13099.32	3	20979.73	4	23277.83	4	28534.32	4	21416.22	4
Liaoning	17807.13	4	27236.93	4	28921.31	4	28945.33	4	26429.94	4
Jilin	9137.27	2	12259.33	3	11695.61	2	8547.83	2	11353.93	2
Heilongjiang	8598.37	2	11771.41	3	16052.23	3	25359.74	4	13856.14	3
Shanghai	11351.33	3	13437.11	3	13690.04	2	13538.12	3	13561.51	3
Jiangsu	21197.52	4	27239.32	4	31070.73	4	32853.74	4	28367.38	4
Zhejiang	12631.63	3	14717.91	3	17406.82	3	14453.42	3	15049.13	3
Anhui	10442.02	3	14220.32	3	17799.02	3	16737.64	3	15097.45	3
Fujian	8803.69	2	12926.64	3	13256.63	2	13813.73	3	12223.94	3
Jiangxi	5789.24	2	9051.01	2	12380.03	2	12385.43	3	10126.82	2
Shandong	33963.52	5	45130.62	5	41919.23	5	37769.34	5	41512.12	5
Henan	20512.44	4	28682.03	4	27676.24	4	21752.62	4	25847.74	4
Hubei	15127.22	4	24449.54	4	22783.73	4	22224.43	4	22109.35	4
Guangdong	15773.21	4	17924.12	3	20916.42	3	20959.24	4	19349.94	4
Guangxi	19189.73	4	26352.43	4	26512.31	4	26956.16	4	25497.32	4
Hunan	7095.99	2	10475.82	2	12281.53	2	11462.82	2	10990.43	2
Hainan	1031.66	1	1961.68	1	3584.97	1	2664.54	1	2198.78	1
Chongqing	6136.07	2	10501.81	2	11692.72	2	9846.43	2	10144.42	2
Sichuan	12175.82	3	22667.32	4	26786.23	4	22484.43	4	21833.73	4
Guizhou	10017.43	3	11025.74	2	14262.73	2	13055.44	3	12781.14	3
Yunnan	10286.71	3	12926.04	3	13387.44	2	15637.51	3	13215.72	3
Shaanxi	8112.34	2	12657.02	3	15644.32	3	13951.47	3	12590.84	3
Gansu	5643.37	2	6650.08	2	8336.09	1	7267.13	2	7202.06	2
Qinghai	1370.82	1	2252.64	1	3556.26	1	3721.23	1	2884.19	1
Ningxia	2919.23	1	4088.43	1	6117.09	1	8437.71	2	5025.63	1
Xinjiang	7348.59	2	10197.63	2	14182.71	2	15698.44	3	12302.44	3

Note: 1, 2, 3, 4, and 5 in the table represent the lowest, lower, medium, higher, and highest emission areas, respectively.

**Fig 3:**
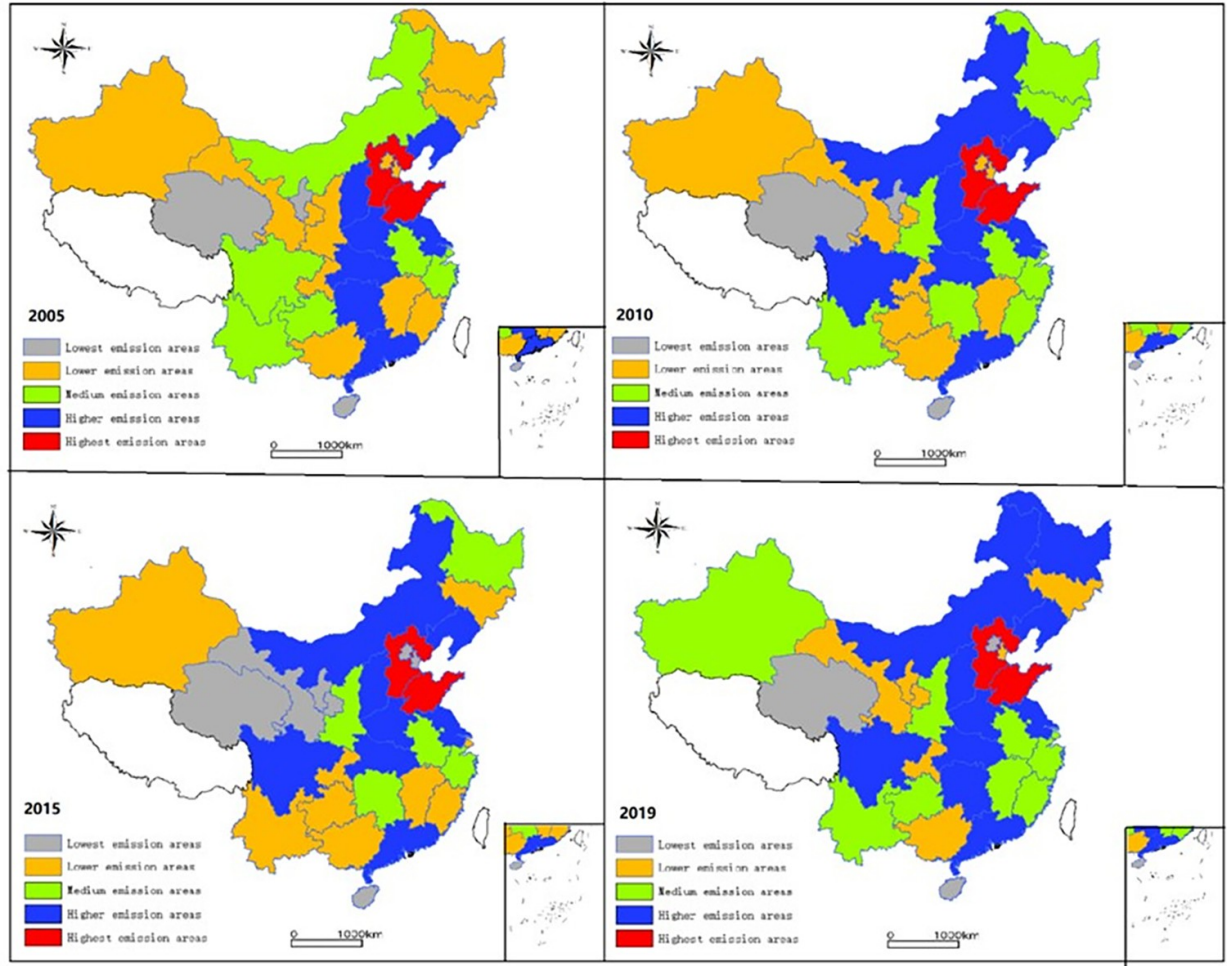
Visualization results of spatial differentiation pattern of regional carbon emissions. (Note: The original picture is from Natural Earth, http://www.naturalearthdata.com).

There is a significant difference between the regions with the highest carbon emissions and the regions with the lowest carbon emissions. From the average carbon emissions of 30 provinces from 2005 to 2019, Hebei has the highest carbon emissions, up to 452.228 million tons, while Hainan has the lowest carbon emissions, only 219.878 million tons. To more clearly analyze the temporal and spatial evolution of regional carbon emissions, Fig 3 is drawn using by GIS visualization method.

As can be seen from Fig 3, the areas with the highest carbon emissions are distributed in Hebei and Shandong in NCER. The higher value areas are distributed in Shanxi, Inner Mongolia, and Henan in ERMRYR, Guangdong in SCER, Jiangsu in ECER, Hunan, and Hubei in ERMRYTR, and Sichuan in SWER. The lowest value area is mainly distributed in Hainan in SCER, Qinghai, and Ningxia in NWER. With time, the growth of carbon emissions in this area is relatively small. It is noteworthy that during the whole study period, Heilongjiang, Fujian, Jiangxi, Shaanxi, and Xinjiang changed rapidly from low carbon emission areas to medium value areas and high carbon emission areas.

The cross-sectional data shows significant changes in the spatial differentiation characteristics of carbon emissions in different regions. In 2005, the carbon emissions of Beijing and Tianjin were in low-value areas, which was closely related to the region’s rapid development during this period. The relatively high-value emission areas include Hebei, Shandong, Shanxi, Liaoning, and other provinces dominated by heavy industry. In 2010, Inner Mongolia and Sichuan entered the high-value area. Jilin, Heilongjiang, Fujian, and Shaanxi also rose from low-value to median areas, and the changes in other provinces were small. In 2015, Beijing’s carbon emissions fell rapidly to the lowest value area, mainly caused by the industrial transfer brought about by the integration of Beijing, Tianjin, and Hebei. As the first batch of carbon market pilots, Shanghai has also dropped from the median area to the lower value area, reflecting the initial results of the national pilot carbon market. In 2019, Heilongjiang, Fujian, Jiangxi, Guizhou, Yunnan, and Xinjiang were upgraded based on the corresponding level compared with 2015, while Beijing was still in the lowest value area, which shows that the integration of Beijing, Tianjin, and Hebei and the introduction of high and new technologies have played a role in carbon emission in this region.

On the whole, carbon emissions show a trend of gradually increasing from west to east, and the carbon emissions of all provinces gradually increase over time. It can be seen from Fig 3 that the carbon emissions of the relatively developed ECER and NCER are generally high. In contrast, the carbon emissions of economically underdeveloped areas such as the NWER and SWER are relatively low.

### 3.2 Analysis of results of temporal and spatial evolution characteristics of regional carbon emission

According to the kernel density function in Section 2.2, this paper analyzes the dynamic evolution characteristics of regional carbon emissions from provincial and regional perspectives and identifies the changing trend of carbon emissions in different regions.

#### 3.2.1 Dynamic analysis of the distribution of provincial carbon emissions in China

**Fig 4:**
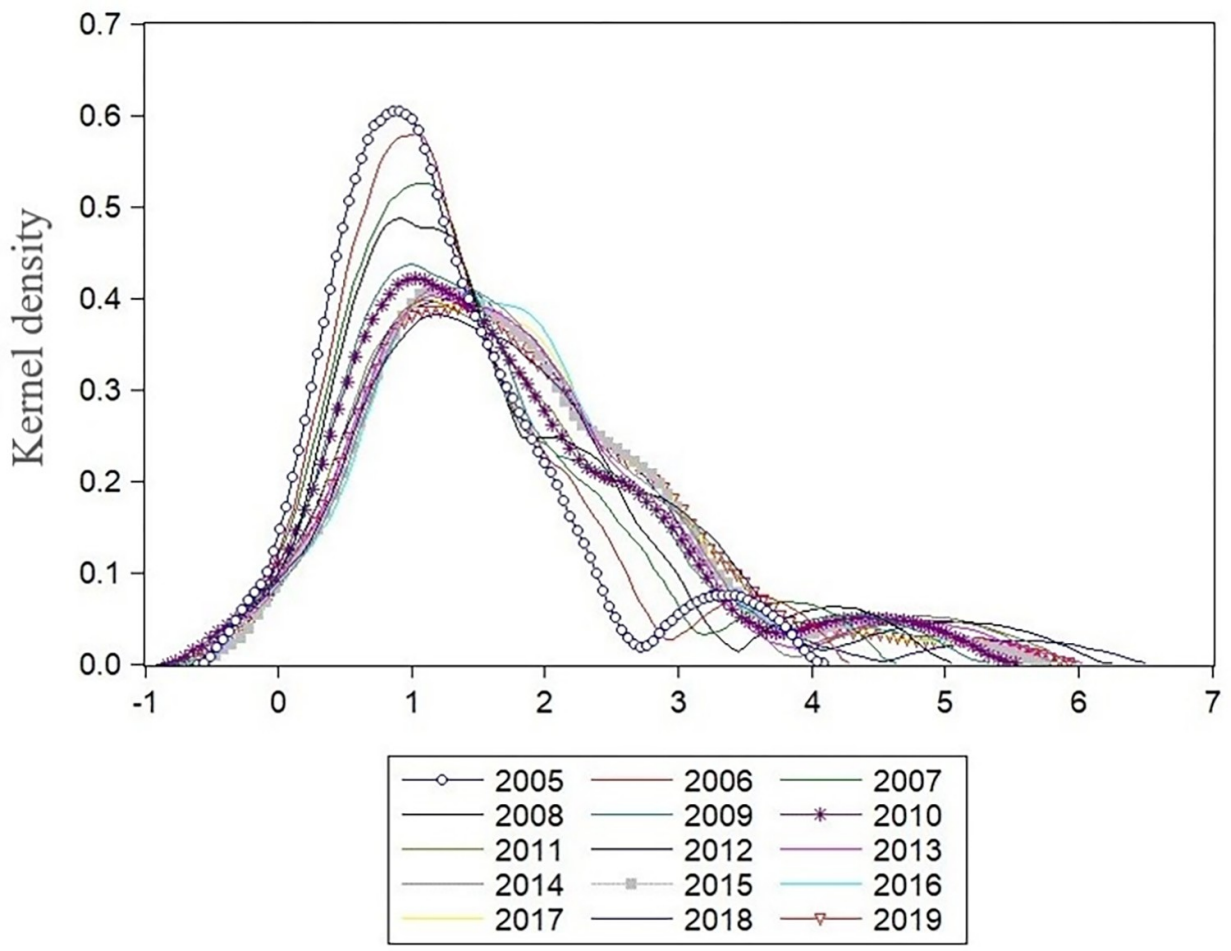
National provincial Kernel density function curve distribution estimation map.

This paper selects 2005–2019 as the research section. It takes 2005, 2010, 2015, and 2019 as the investigation time points, respectively, to make a dynamic analysis of the regional differences in carbon emissions of 30 provinces in China from the national and provincial levels, as shown in Fig 4. From the perspective of the position of the center of gravity, the trend of the right deviation of the core density curve in the study period from 2005 to 2019 is significant, reflecting the rapid growth of carbon emissions in 30 provinces of China. The two-tailed extension of the kernel density curve shifts to the right-side year by year. The extension of the right side is significantly greater than that of the left side, which indicates that the volatility of the high-value area of local carbon emission is relatively large. From the peak value, the peak height in 2005 was the highest, and the peak moved from left to right, but the moving range was relatively small. However, the peak height decreased significantly, indicating that the aggregation phenomenon of provincial low-value areas of carbon emissions still exists, and the regional difference is gradually increasing. From the perspective of shape and flexibility, as time goes on, the annual curve kurtosis evolves from peak distribution to wide peak distribution on the whole, and the change range gradually increases, showing a widening trend, which shows that the provincial carbon emission tends to vary significantly. This shows the rapid growth of carbon emissions and the direction of continuous expansion of regional carbon emission differences. Therefore, targeted and regional environmental restraint policies need to be formulated for high-value and low-value areas of carbon emissions.

#### 3.2.2 Dynamic evolution analysis of carbon emission in China’s eight economic regions

**Fig 5:**
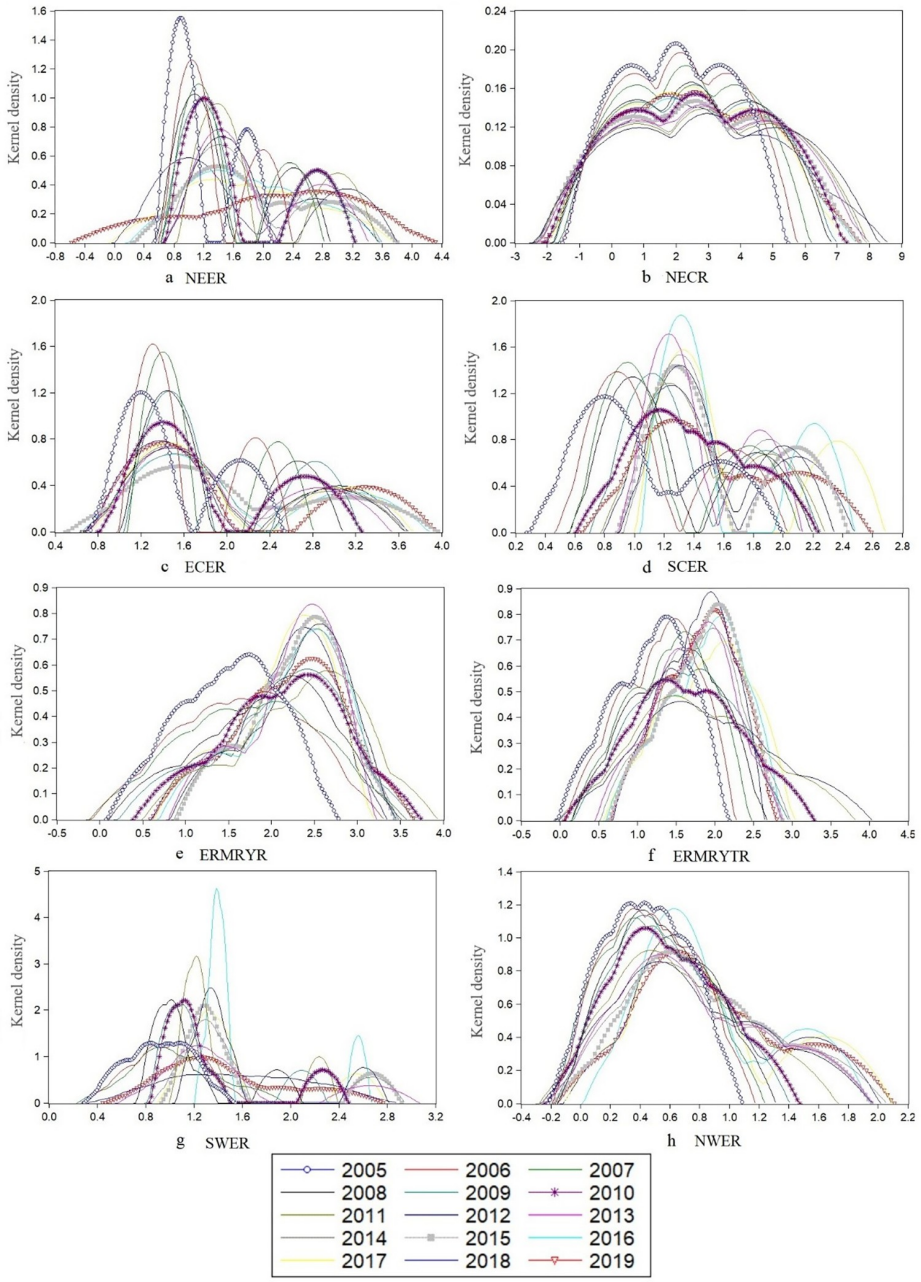
Temporal and spatial evolution trend of carbon emissions in China’s eight economic regions.

This paper analyzes the dynamic evolution characteristics of carbon emissions in China’s eight economic regions and draws Fig 5. As can be seen from Fig 5, on the whole, the fluctuation and variation of the kernel density curve of carbon emission in the eight economic regions are significantly different. Besides the ERMRYTR, the height of the wave crest in the other areas decreased to varying degrees. Overall, the kernel density curve of the eight economic regions showed a trend of moving to the right from 2005 to 2019.

From the point of view of the carbon emission curve, the number of nuclear emission curves increases to the right. The kernel emission curves decrease in the region with high carbon density. In NEER (Fig 5A), except for a certain degree of left shift in 2018, the overall right shift trend of the kernel density curve in Northeast China from 2005 to 2019 is significant. The kernel density curve of the NCER (Fig 5B) shows a slight right shift trend. The other six regions (Fig 5C–5H) showed a right-shift trend. This indicates that during the study period, the low-value area of carbon emission corresponding to the kernel density curve of each region is declining, and the high-value area is rising, indicating that the overall regional carbon emission is increasing.

From the morphological point of view, in most years of 2005–2019, the kernel density curves of NEER (Fig 5A), ECER (Fig 5C), SCER (Fig 5D), and SWER (Fig 5G) showed an "M" shape with the prominent peak. A secondary rise coexisted, indicating an apparent two-level differentiation of carbon emission in these areas. Except for the NEER, the wave peaks of the other three regions fluctuate considerably, and the average year of 2005 is not the highest peak. The peak heights of the NCER (Fig 5B) and the NWER (Fig 5H) were the highest in 2005, but the decline of peak heights in these two areas was relatively small. Carbon emissions in the ERMRYR (Fig 5E) and the ERMRYTR (Fig 5F) increased in 2012 and 2018.

From the perspective of flexibility, the right tail corresponding to the nuclear density curve in most areas is longer than the left, changing from peak to wide peak distribution. The change range is relatively increased. The ductility shows a trend of continuous widening. Among them, the NEER (Fig 5A) shows a stable decline from the peak height over time and changes from the peak distribution in 2005 to the wide peak distribution in 2019, which indicates that the regional carbon emission tends to change significantly, and the growth rate of high-value areas of carbon emission is fast. The kurtosis of nuclear density in the NCER (Fig 5B) gradually widened, indicating that the right tail on both sides is longer than the left tail, on the whole, suggesting that the modified area’s carbon emissions showed the phenomenon of low-value area accumulation during the study period. The ductility analysis of other regions is consistent with the analysis methods of the above two areas, which will not be repeated here.

To sum up, it can be seen that the distribution of the kernel density curve in the eight economic regions is quite different, and there are significant differences in both the position of the center of gravity of the kernel density curve and the shape and flexibility of the kernel density curve. This shows substantial differences in regional carbon emissions due to significant differences in resource endowment and energy consumption structure in different development stages.

### 3.3 Analysis of regional carbon emission differences

According to Formulas (4–6), the Theil index of the overall inter-regional and intra-regional difference of the eight economic regions from 2005 to 2019 is calculated, as shown in Table 3.

**Table 3:** Theil index of regional carbon emission difference and its decomposition results.

Region	Index	2005	2006	2007	2008	2009	2010	2011	2012	2013	2014	2015	2016	2017	2018	2019
NEER	α	1.50	1.53	1.56	1.47	1.50	1.44	1.40	1.22	0.84	0.78	0.62	0.46	0.74	1.79	0.76
	β	2.62	3.03	3.45	2.83	3.06	3.06	3.21	3.26	3.02	2.94	3.18	3.52	3.24	1.88	4.49
	γ	4.12	4.56	5.01	4.30	4.56	4.50	4.61	4.48	3.85	3.73	3.80	3.99	3.98	3.67	5.25
NCER	α	5.49	5.58	5.43	5.46	5.46	5.82	6.43	6.07	6.89	6.27	6.11	6.19	6.12	7.78	5.79
	β	2.86	2.98	3.22	3.38	3.19	3.55	3.64	3.66	3.56	3.25	2.75	3.43	2.34	4.44	2.97
	γ	8.35	8.56	8.65	8.84	8.64	9.36	10.08	9.74	10.45	9.52	8.86	9.62	8.47	12.23	8.75
ECER	α	0.25	0.16	0.27	0.32	0.39	0.59	0.77	0.77	0.71	0.80	0.86	0.91	0.95	1.12	1.16
	β	5.57	5.67	5.76	5.86	5.91	5.92	5.87	5.94	5.58	5.69	5.57	5.23	5.41	5.53	5.53
	γ	5.82	5.82	6.04	6.18	6.30	6.51	6.64	6.72	6.29	6.49	6.43	6.14	6.37	6.66	6.69
SCER	α	0.93	0.86	0.81	0.87	0.94	1.15	1.17	1.15	1.20	1.19	1.22	2.89	0.96	0.91	0.94
	β	4.68	4.79	4.84	4.83	4.74	4.85	4.92	4.95	4.93	4.80	4.74	5.72	4.72	4.71	4.66
	γ	5.61	5.65	5.65	5.70	5.68	6.00	6.09	6.09	6.13	5.98	5.96	8.61	5.68	5.62	5.60
ERMRYR	α	5.21	5.27	5.29	5.78	5.74	5.45	5.73	5.88	6.26	6.03	6.28	6.74	6.88	7.82	8.26
	β	4.94	5.01	4.99	5.16	5.16	5.25	5.40	4.72	4.88	4.93	4.92	5.56	4.57	4.71	4.73
	γ	10.16	10.27	10.28	10.94	10.91	10.70	11.13	10.60	11.14	10.96	11.20	12.31	11.46	12.52	12.99
ERMRYTR	α	1.61	1.39	1.27	1.35	1.23	1.52	1.71	1.71	0.73	0.68	0.39	0.32	0.23	0.33	0.41
	β	0.05	0.01	0.02	0.23	0.43	0.64	0.70	0.79	1.38	1.49	1.38	1.15	1.41	2.05	2.11
	γ	1.65	1.40	1.29	1.58	1.66	2.16	2.42	2.50	2.11	2.17	1.77	1.47	1.64	2.38	2.52
SWER	α	3.99	3.77	3.33	2.56	2.56	2.43	2.48	2.68	2.75	2.82	2.68	2.83	3.07	4.21	3.21
	β	1.42	1.27	1.11	1.52	1.68	1.56	1.15	1.58	1.12	1.27	0.86	1.27	0.57	0.56	0.08
	γ	5.40	5.04	4.45	4.08	4.24	3.98	3.63	4.26	3.87	4.10	3.54	4.10	3.64	4.78	3.29
NWER	α	0.85	0.98	0.94	1.15	1.12	1.21	1.34	1.23	1.25	1.31	1.51	1.86	1.91	1.96	1.99
	β	2.92	2.96	2.74	3.05	3.06	3.37	3.78	4.48	5.49	5.83	6.17	7.04	7.32	7.49	7.34
	γ	3.77	3.94	3.68	4.21	4.18	4.58	5.12	5.71	6.74	7.14	7.68	8.90	9.23	9.46	9.33

Note: α, β, and γ represent the Theil index of intra-regional differences, inter-regional differences, and absolute differences.

From the value of α, the intra-regional difference between the NCER and the ERMRYR is the largest. The regional differences in the NCER show a fluctuating upward trend, and the regional differences in the ERMRYR show a slow upward trend. Among them, the regional carbon emission differences in the NEER, the ERMRYTR, and the SWER show a fluctuating downward trend, which indicates that these regions have achieved phased results in carbon emission reduction. The difference in carbon emission in the ECER has the most significant change, rising from 0.25 in 2005 to 1.16 in 2019, increasing 458%.

From the value of β, there are significant differences in inter-regional carbon emissions among the eight economic regions. The ECER and the NWER are the regions with the most considerable difference in carbon emissions. The NWER has the most prominent change range, rising from 2.92 in 2005 to 7.34 in 2019, about 151.5%. The regional differences in the ERMRYTR, the NEER, and the NCER showed a fluctuating upward trend, and the other four regions showed a slight downward trend.

From the value of γ, there are apparent differences in the overall carbon emissions in different regions. Among them, the overall Theil index in the ERMRYR is the largest and shows a fluctuating upward trend, rising from 10.16 in 2005 to 12.99 in 2019. The SCER and SWER decreased slightly, from 5.61 and 5.40 in 2005 to 5.60 and 3.29 in 2019. Secondly, the NCER also increased slightly from 8.35 in 2005 to 8.75 in 2019, indicating that the region needs to make more efforts to reduce carbon emissions in the future.

**Fig 6:**
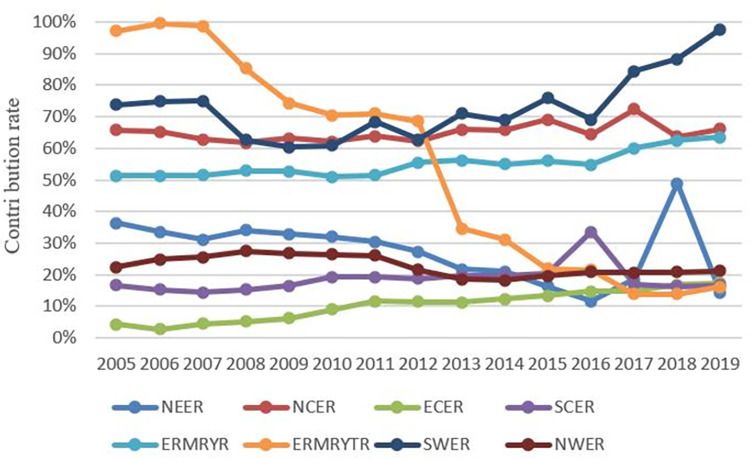
The contribution rate of intra-regional differences.

To further analyze the impact of inter-regional and intra-regional differences on the overall differences, this paper calculates the contribution rate of inter-regional and intra-regional differences to the overall regional differences, as shown in Fig 6. From the contribution rate change of intra-regional differences, the ERMRYTR decreased the most during the study period, from 97.08% in 2005 to 16.12% in 2019. It is the region with the most significant decline in the contribution rate of eight regions, with a decrease of 83.40%. The second is the NEER, which decreased from 36.338% in 2005 to 14.49% in 2019, decreasing by 21.89%. The change in NWER is relatively small, and the intra-regional contribution rate declined by only 1.2% during the study period. The contribution rates of intra-regional differences in the other five regions vary. The ECER has the most remarkable change, rising from 4.26% in 2005 to 17.35% in 2019, rising 13.1 percentage points. The intra-regional contribution rate in the ERMRYR accounts for more than half. The intra-regional difference is the main contributor to the regional carbon emission difference and shows a rising trend during the study period, from 51.34% in 2005 to 63.56% in 2019, with 23.81%. The contribution of intra-regional differences in the SWER is enormous. In 2005, the region’s contribution rate of intra-regional differences was as high as 73.79%. It fluctuated and rose during this period, reaching the maximum value of 97.58% in 2019. The difference in intra-regional carbon emissions has a decisive impact on the region’s difference in carbon emissions. However, the contribution rate of intra-regional differences in the NCER and the SCER changes is relatively minor, increasing by 0.5% and 0.4%, respectively. However, the difference is that the contribution rate of intra-regional differences in the NCER has been more than 60% over the years, indicating that the intra-regional differences have a significant impact on the difference in carbon emissions in this area, while the contribution rate of intra-regional differences in the SCER is mostly less than 20%. It shows that the impact of intra-regional differences on the overall carbon emission difference is limited.

**Fig 7:**
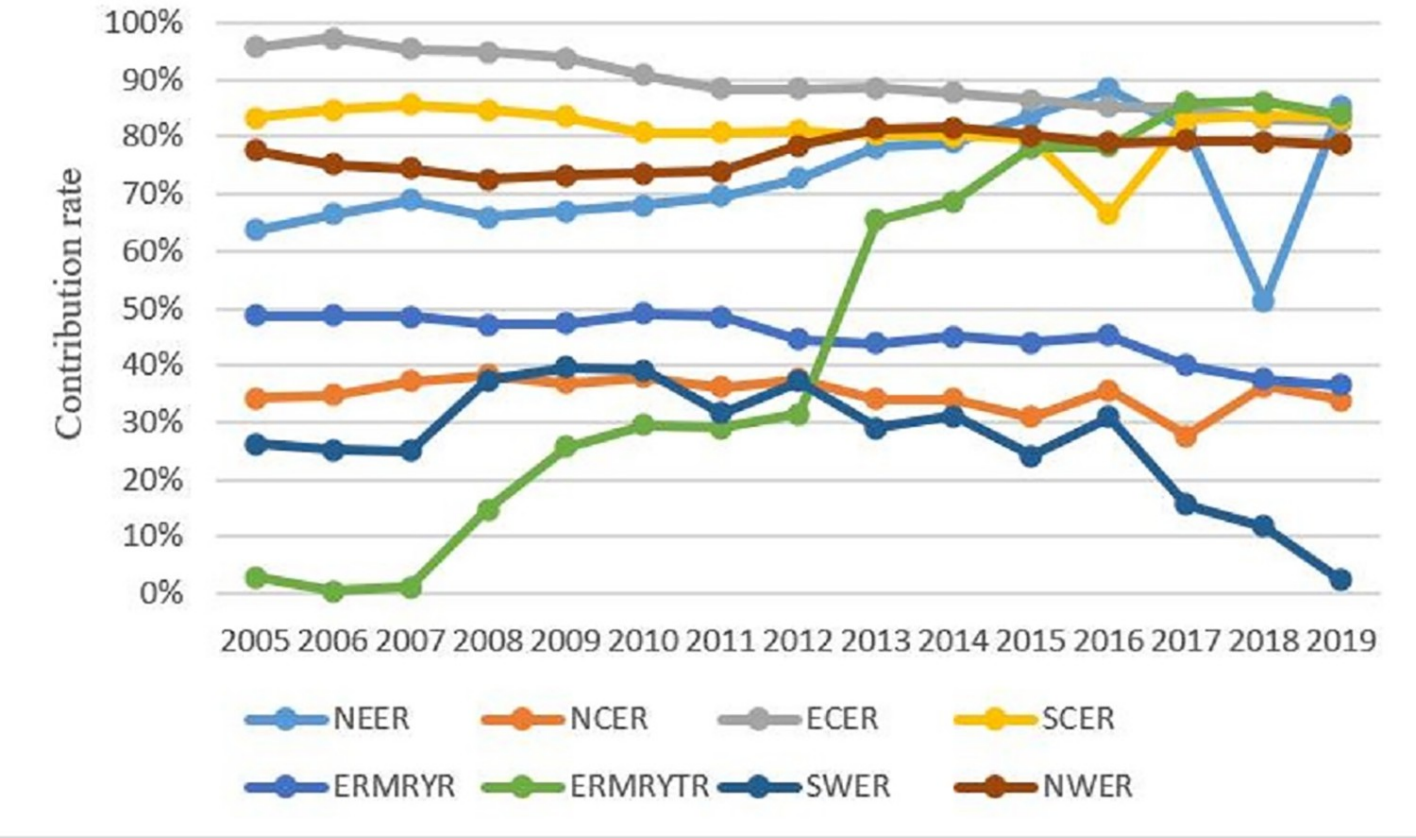
The contribution rate of inter-regional differences.

Similarly, it can be seen from Fig 7 that the change in the inter-regional difference contribution rate in corresponding regions corresponds to the changing trend of the intra-regional difference contribution rate. The regions with a relatively stable contribution rate of inter-regional differences accounting for more than 50% are the ECER, SCER, NWER, and NEER during the study period. The contribution rate of inter-regional differences in the ERMRYTR increased significantly in 2013, from 31.45% in 2012 to 65.41% in 2013. Since then, it has grown steadily and gradually increased to 83.88% in 2019, becoming the region with the most considerable contribution to interregional differences.

### 3.4 Analysis of decoupling state results in different regions

Due to space constraints, this paper divides the decoupling state analysis of the eight economic regions into four stages: 2005–2010, 2010–2015, 2015–2019, and 2005–2019 (Table 4). In the whole study period from 2005 to 2019, only Jilin and Beijing showed strong decoupling, and the other 28 provinces showed weak decoupling. Overall, the dependence of GDP growth on fossil energy in most parts of the country is declining. Economic growth is developing from extensive to intensive, and the overall economic growth model is resource-friendly.

**Table 4:** One-dimensional decoupling state for China’s eight economic regions over 2005–2019.

Regions	2005–2010			2010–2015			2015–2019			Mean value (2005–2019)		
	ΔGDP%	ΔCO2%	EP	ΔGDP%	ΔCO2%	EP	ΔGDP%	ΔCO2%	EP	ΔGDP%	ΔCO2%	EP
Liaoning	69.69	52.96	0.76	41.74	6.18	0.15	16.56	0.08	0.01	180.35	62.55	0.35
Jilin	68.64	34.17	0.50	47.25	-4.60	-0.10	20.48	-26.91	-1.31	199.18	-6.45	-0.03
Heilongjiang	64.43	36.90	0.57	43.96	36.37	0.83	20.27	57.98	2.86	184.70	194.94	1.06
**NEER**	**67.80**	**44.24**	**0.65**	**43.48**	**10.54**	**0.24**	**18.49**	**10.91**	**0.59**	**185.28**	**76.84**	**0.41**
Beijing	70.81	6.00	0.08	43.96	-5.67	-0.13	29.25	-8.18	-0.28	217.83	-8.20	-0.04
Tianjin	86.84	47.64	0.55	59.69	-16.73	-0.28	18.77	54.22	2.89	254.37	89.60	0.35
Hebei	54.12	31.71	0.59	47.55	10.61	0.22	29.25	2.57	0.09	193.93	49.44	0.25
Shandong	67.10	32.88	0.49	55.39	-7.12	-0.13	28.99	-9.90	-0.34	234.95	11.20	0.05
**NCER**	**66.39**	**31.03**	**0.47**	**51.61**	**-0.35**	**-0.01**	**28.01**	**0.14**	**0.00**	**222.93**	**30.75**	**0.14**
Shanghai	70.29	18.37	0.26	43.96	1.88	0.04	29.49	-1.11	-0.04	217.43	19.27	0.09
Jiangsu	88.48	28.50	0.32	58.26	14.07	0.24	30.58	5.74	0.19	289.49	54.99	0.19
Zhejiang	75.29	16.52	0.22	48.43	18.27	0.38	32.55	-16.97	-0.52	244.88	14.42	0.06
**ECER**	**80.07**	**22.61**	**0.28**	**52.09**	**12.23**	**0.23**	**30.96**	**-2.13**	**-0.07**	**258.66**	**34.67**	**0.13**
Fujian	90.98	46.83	0.51	66.34	2.55	0.04	36.42	4.20	0.12	333.40	56.91	0.17
Guangdong	80.52	37.33	0.46	50.76	0.61	0.01	31.07	1.67	0.05	256.72	40.47	0.16
Hainan	83.04	90.15	1.09	57.50	82.75	1.44	28.75	-25.67	-0.89	271.18	158.28	0.58
**SCER**	**82.89**	**42.09**	**0.51**	**54.53**	**5.12**	**0.09**	**32.32**	**0.19**	**0.01**	**273.97**	**49.64**	**0.18**
Shanxi	57.49	26.26	0.46	41.47	5.39	0.13	25.75	-2.24	-0.09	180.17	30.08	0.17
Inner Mongolia	114.70	60.16	0.52	58.70	10.95	0.19	23.15	22.58	0.98	319.64	117.83	0.37
Henan	83.36	39.83	0.48	58.67	-3.51	-0.06	34.04	-21.40	-0.63	289.96	6.05	0.02
Shaanxi	88.15	56.02	0.64	65.52	23.60	0.36	32.79	-10.82	-0.33	313.53	71.97	0.23
**ERMRYR**	**84.43**	**42.33**	**0.50**	**57.14**	**6.47**	**0.11**	**30.52**	**-3.20**	**-0.10**	**278.26**	**46.69**	**0.17**
Anhui	87.01	36.18	0.42	65.10	25.17	0.39	36.92	-5.96	-0.16	322.76	60.29	0.19
Jiangxi	85.87	56.34	0.66	64.40	36.78	0.57	39.09	0.04	0.00	325.03	113.93	0.35
Hubei	92.02	61.63	0.67	66.57	-6.81	-0.10	34.79	-2.45	-0.07	331.14	46.92	0.14
Hunan	93.37	13.64	0.15	64.37	16.69	0.26	35.29	0.20	0.01	330.01	32.88	0.10
**ERMRYTR**	**90.06**	**39.28**	**0.44**	**65.20**	**12.54**	**0.19**	**36.20**	**-2.13**	**-0.06**	**327.63**	**53.41**	**0.16**
Guangxi	72.46	47.63	0.66	57.80	17.24	0.30	29.73	-6.67	-0.22	253.06	61.54	0.24
Chongqing	101.74	71.15	0.70	82.80	11.34	0.14	36.33	-15.79	-0.43	402.77	60.47	0.15
Sichuan	90.11	86.17	0.96	65.42	18.17	0.28	35.17	-16.06	-0.46	325.08	84.66	0.26
Guizhou	81.11	10.07	0.12	79.78	29.36	0.37	43.88	-8.46	-0.19	368.46	30.33	0.08
Yunnan	74.65	25.66	0.34	67.89	3.57	0.05	40.12	16.81	0.42	310.86	52.02	0.17
**SWER**	**85.19**	**47.87**	**0.56**	**69.16**	**16.00**	**0.23**	**36.22**	**-7.55**	**-0.21**	**326.74**	**58.57**	**0.18**
Gansu	70.61	17.84	0.25	66.13	25.35	0.38	25.49	-12.82	-0.50	255.66	28.77	0.11
Qinghai	77.30	64.33	0.83	63.77	57.87	0.91	31.32	4.64	0.15	281.30	171.46	0.61
Ningxia	73.72	40.05	0.54	58.64	49.62	0.85	31.08	37.94	1.22	261.24	189.04	0.72
Xinjiang	65.28	38.77	0.59	65.60	39.08	0.60	30.33	10.69	0.35	256.72	113.62	0.44
**NWER**	**69.09**	**34.18**	**0.49**	**64.85**	**38.83**	**0.60**	**28.82**	**9.11**	**0.32**	**259.09**	**103.24**	**0.40**

**Fig 8:**
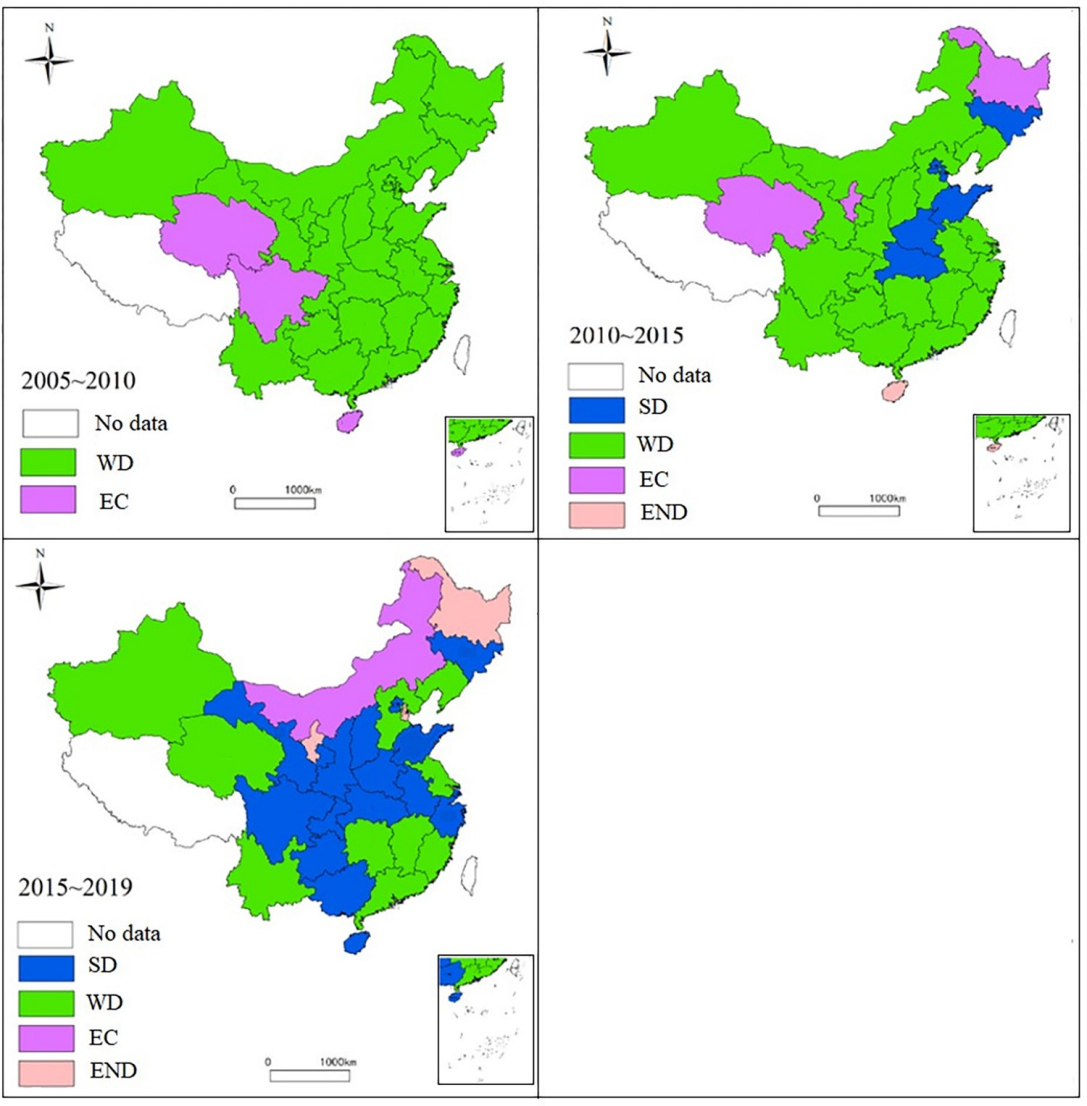
Decoupling pattern and type distribution of carbon emissions and economic growth. (Note: The original picture is from Natural Earth, http://www.naturalearthdata.com).

To observe the change in the decoupling state of carbon emission in each region, Fig 8 is drawn to observe the evolution of the decoupling state in different periods.

It can be seen that the relationship between carbon dioxide emissions and GDP in most regions from 2005 to 2010 is in a weak decoupling state. Hainan, Sichuan, and Qinghai are in an expansionary connection state. Only Hainan has a decoupling elasticity value greater than 1, which indicates that the growth of carbon emissions in each region is slower than that of GDP. Moreover, the decoupling elasticity value in ECER is the most minor, only 0.28, and this shows that while achieving the same economic growth, the region emits relatively less carbon dioxide. From 2010 to 2015, more than half of the regions were still weakly decoupled, while Jilin, Beijing, Tianjin, Shandong, Henan, and Hubei are strongly decoupled, indicating that the economic growth mode of the above regions is resource-saving and environment-friendly. Hainan is in an expansionary negative decoupled state, and the growth of carbon emission is 1.44 times that of GDP. It shows that the region’s economic development depends heavily on fossil energy at this stage, which is an extensive economic growth model. From 2015 to 2019, more than half of the regions showed strong decoupling, and Heilongjiang, Tianjin, and Ningxia were in expansionary negative decoupling, which was closely related to the high dependence on fossil energy consumption in the process of seeking development, and the other third showed weak decoupling.

### 3.5 Analysis of numerical simulation results

**Fig 9:**
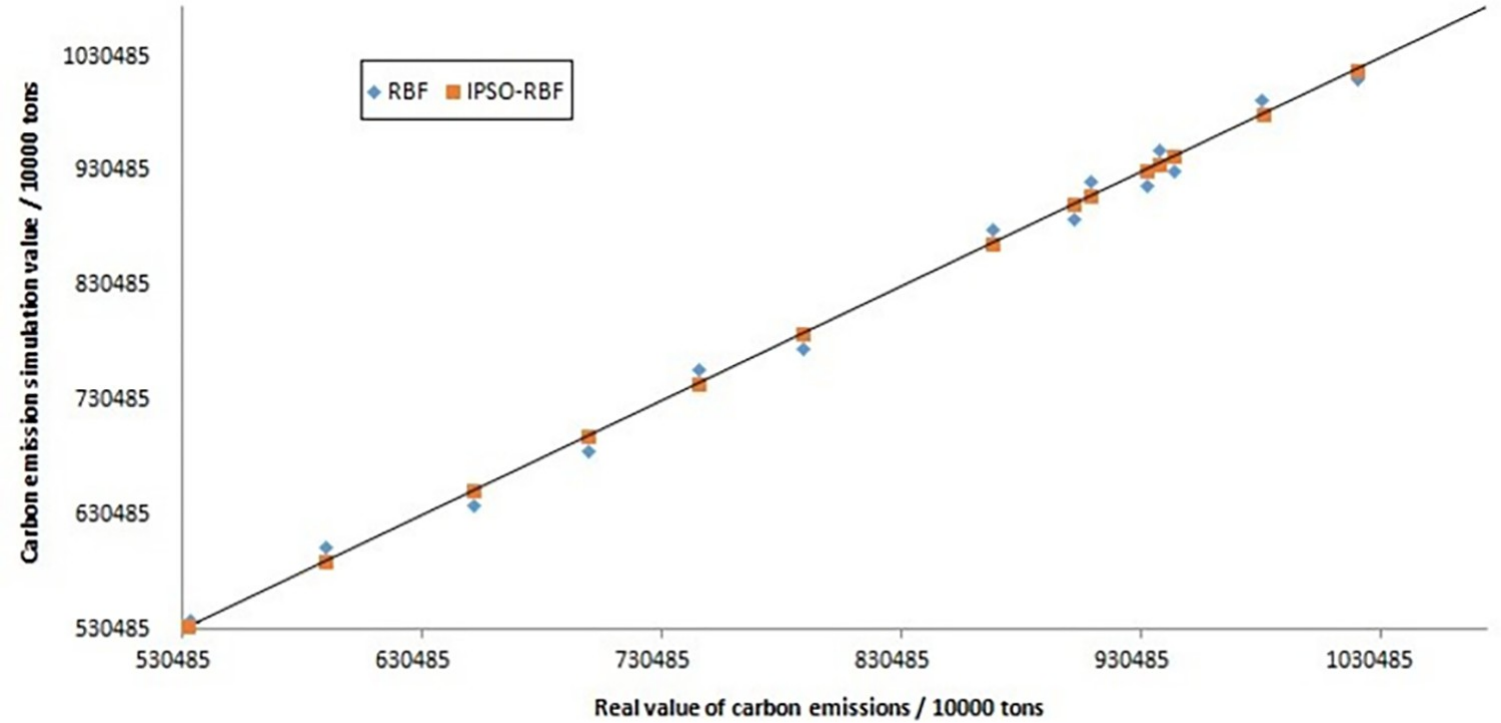
Comparison of simulation results between IPSO-RBF model and RBF neural network.

Due to the relatively slow updating of China’s carbon emission data, the previous prediction models cannot meet the actual demand. In order to more accurately predict the relevant data of future carbon emissions, such as the carbon emissions of provinces and eight economic regions under the dual carbon goals of 2030 and 2060, this paper introduces different carbon emission prediction models for comparative analysis. We take the national carbon emission data from 2005 to 2015 as the training array and simulate the prediction results from 2016 to 2019. The comparison of simulation results is shown in Fig 9. A large number of data statistics show that the prediction results of the IPSO-RBF neural network model are closer to the actual value. The diagonal in the figure and the prediction points are more compact; The diagonal deviation of the RBF neural network model is larger than the actual value, which is more scattered. This shows that the prediction result of the IPSO-RBF model is better than RBF neural network. The improved particle swarm optimization algorithm optimizes RBF neural network, making the prediction result more stable and accurate.

The root means square error (RMSE), mean relative error (MRE), mean absolute error (MAE), standard deviation (SD), and coefficient of determination (R^2^) are used to measure the error and accuracy of the two prediction models, as shown in Table 4. RMSE, MRE, MAE, and SD reflect the deviation of prediction data from the actual value. The smaller the value, the smaller the prediction error and the higher the prediction accuracy; Therefore, it is evident that the RMSE, MRE, Mae, and SD values of the IPSO-RBF model are smaller than those of the RBF neural network, indicating that the prediction error of IPSO-RBF model is less than that of RBF neural network.

The more the coefficient of determination (R^2^) tends to 1, the higher the degree of interpretation of the independent variable to the dependent variable [35]. They take the actual value as the x-axis and the predicted value as the y-axis. When R^2^ tends to 1, the higher the reference value of equation y = x, indicating that the expected value tends to be the actual value. Therefore, it is evident that the R^2^ of IPSO-RBF and RBF neural network models are above 0.9 (Table 5), but the former value is greater than that of the latter, indicating the goodness of fit in the IPSO-RBF model is better. Therefore, the IPSO-RBF model is more suitable for predicting carbon emissions and related indicators. This model should predict carbon emissions in the whole country and different regions in future research.

**Table 5:** Comparative analysis of error indexes of two neural network models.

Indicator	Model	RMSE	MRE%	MAE%	SD	R^2^
Carbon emission	RBF	31256.635	0.434	3.873	93642.361	0.956
	IPSO-RBF	8323.034	0.272	0.956	73243.652	0.989

## 4. Conclusion and implications

Based on the complete analysis of carbon emission characteristics in China’s eight economic regions, this paper explores carbon emission’s temporal and spatial evolution characteristics in different regions, the leading causes of carbon emission differences, and the evolution law of regional carbon emission decoupling states. Through comparative analysis, a more accurate carbon emission prediction method is found. The main conclusions of this paper are as follows:

1. There are significant differences in carbon emissions among China’s eight economic regions. The high-value areas of carbon emissions are mainly concentrated in Shandong and Hebei in NCER. In contrast, the low-value areas are Hainan, Qinghai, and Ningxia, and the growth over time is relatively small. On the whole, carbon emissions are gradually increasing from west to east. The dynamic characteristics of national carbon emissions slowly evolve from peak to broad peak, which indicating that the difference between high-value and low-value areas of provincial carbon emissions is expanding. The overall kernel density curve shifts to the right. This shows that that the high carbon emission area fluctuation is relatively large. From the regional perspective, there are significant differences in the dynamic evolution trend of carbon emissions in different regions. Among them, from the position of the center of gravity, the gap between high-value and low-value areas of carbon emissions in most regions is increasing. From the morphological point of view, most areas show the coexistence of double or multi-peaks. There are also significant differences in scalability, which indicates that there are significant differences in regional carbon emissions due to significant differences in resource endowments and energy consumption structures at different development stages.
2. The decomposition results of regional carbon emission differences show that the reasons for the overall differences of carbon emissions in different regions are different. Inter-regional differences are the leading causes of regional carbon emission differences, and the overall Theil index of ERMRYR is the highest. Regarding the contribution rate of intra-regional differences, ERMRYTR decreased the most. In terms of the time change of the decoupling state, the regions with strong decoupling are increasing, which shows that the dependence of GDP growth on fossil energy is decreasing in most regions. And the model of economic growth is developing from extensive to intensive and resource-friendly.
3. From the comparison of regional carbon emission prediction models, it can be seen that the IPSO-RBF neural network model is more feasible. And the RMSE, MRE, MAE, and SD values are less than those of the RBF neural network. The value of R^2^ is greater than that of the RBF neural network. The improved particle swarm optimization algorithm significantly optimizes the RBF neural network. The prediction result of the IPSO-RBF neural network model is more stable, with minor errors and higher accuracy. It is more suitable for predicting regional carbon emissions and related indicators. Therefore, in realizing China’s double carbon goal, IPSO-RBF neural network model is more suitable for predicting carbon emissions and other relevant indicators in various regions due to its higher accuracy and training speed.

Through the above research, we get the following main implications:

1. There are significant differences in the spatio-temporal evolution trends and differences in regional carbon emissions. On the whole, the eastern regions with more developed economies have relatively large carbon emissions. On the contrary, the less developed regions have relatively small carbon emissions. The causes of significant differences in carbon emissions in different regions are also significantly different. Furthermore, the decoupling relationship between carbon emissions and economic development in different regions has also significantly changed over time. Therefore, on the whole, when formulating regional carbon emission reduction countermeasures, we should not cut across the board but should formulate corresponding emission reduction measures according to the characteristics of different regions’ carbon emissions. First, targeted emission reduction measures should be taken at the regional level, and the overall regional emission reduction targets should be broken down into different provinces based on the respective characteristics of different provinces in different regions. Through this national—regional—provincial distribution method, we can achieve a proper decomposition of carbon emission reduction targets and help to achieve China’s dual carbon goals.
2. Based on thoroughly studying the characteristics of carbon emissions in different provinces and regions and finding targeted carbon emission reduction targets in different regions, we should further explore regional carbon emission reduction issues in the next decade or even longer in combination with China’s recent dual carbon targets. Therefore, the prediction of future provincial and regional carbon emissions will become the focus of future research. Based on comprehensively analyzing the prediction effects of different models, the carbon emission prediction model with the best goodness of fit and the most stable and accurate prediction results were found. From the experimental data, it can be concluded that the IPSO-RBF model has the best prediction effect, so it can be given priority in predicting future provincial, regional, and national carbon emissions.
